# The psychological typhoon eye effect during the COVID-19 outbreak in China: the role of coping efficacy and perceived threat

**DOI:** 10.1186/s12992-020-00626-8

**Published:** 2020-10-27

**Authors:** Li Zhang, Min Ma, Danfeng Li, Ziqiang Xin

**Affiliations:** Central University of Finance and Economics, 39 Xueyuan South Road, Beijing, China

**Keywords:** Exposure to COVID-19, Mental health problems, Coping efficacy, Perceived threat, Psychological typhoon eye

## Abstract

**Background:**

The influence of COVID-19 on mental health problems has received considerable attention. However, only a few studies have examined the relationship between exposure to COVID-19 and mental health problems, and no empirical study has tested the mechanisms between them.

**Methods:**

We conducted a survey in 31 provinces of China during 3–13 March 2020 to test the effect of the exposure level on mental health problems. Our sample comprised 2987 participants who reported their perceived threat, coping efficacy, mental health problems and other demographic variables. Multiple mediators path analysis was used in the data analysis.

**Results:**

The results showed that the level of exposure to COVID-19 in China was negatively associated with mental health problems, which confirmed the “Psychological Typhoon Eye” effect. Further analyses indicated that both perceived threat and coping efficacy partially mediated the relationship between them. However, coping efficacy explained the “Psychological Typhoon Eye” effect. Perceived threat mediated the positive relationship between exposure level and mental health problems.

**Conclusion:**

This study detected the psychological typhoon eye effect and demonstrated the mediating role of coping efficacy and perceived threat between exposure to COVID-19 and mental health problems. Our findings suggest that policy makers and psychological workers should provide enough psychological services to low-risk areas as the high-risk areas. An important means of alleviating mental health problems is to improve coping efficacy.

## Background

The recent outbreak of coronavirus disease (COVID-19) in China and worldwide is a major public health emergency of international concern and has been characterized by the World Health Organization as one of the most challenging outbreaks to date. As of 11 June 2020, around 7.2 million confirmed cases globally, 84,652 in China, with 413,372 deaths (5.68%) had been reported by the WHO. Reviews in the field of exposure to COVID-19 and mental health problems have called for research to test the relationship between them and to identify the mechanism underlying this relationship [26, 51, 52]. The present study examined the risk perception factors that may explain how the level of exposure to COVID-19 in China contributes to mental health problems.

Many organizations and researchers have highlighted concerns about mental health problems in affected communities. Major public health emergencies, such as the severe acute respiratory syndrome coronavirus (SARS-CoV) in 2002, the Middle East respiratory syndrome coronavirus (MERS-CoV) in 2012, the West Africa Ebola virus disease (EVD) pandemic in 2013–2016, and the global COVID-19 pandemic typically lead to widespread fear and panic. For example, a critical review indicated that SARS survivors consistently reported high rates of emotional distress persisting for years [10]. During the West Africa EVD pandemic, there were increasing risks for new-onset psychological distress and psychiatric disorders [37]. Psychosocial effects include adjustment disorders, symptoms of PTSD, anxiety, and depression [18, 22, 32]. To date, several studies have indicated the influence of COVID-19 on mental health problems. For instance, the pandemic has burdened a major psychological stress on the medical workforce [28] and could cause distress and leave many people vulnerable to mental health problems and suicidal behavior [13]. Thus, the influence of COVID-19 on mental health problems cannot be ignored. To manage psychological sequelae, it is important to detect the antecedents of mental health problems.

The antecedents of mental health problems during public health emergencies include many factors, such as the exposure level, quarantine, social support, social rejection or isolation, and the news media conveying risk-elevating messages about the public health crisis [2, 27, 35, 42]. Specific to COVID-19, some studies have revealed that risk perception, health anxiety, social media use and more media engagement are predicators to mental health problems [1, 4, 31]. Among these factors, an obvious objective variable is the extent to which people are exposed to emergencies and disasters in their daily life, i.e., the exposure level. According to the ripple effect found in the seminal study by Slocvic (1987), the impact of an unfortunate event decays gradually as ripples spread outward from the center; the closer people are to the center (i.e., the higher the exposure level), the stronger their mental distress is.

However, a few studies have found that this is not the case [25, 51]. Studies have found that proximity to the center of the epidemic or devastated area was negatively related to anxiety levels [51], epidemic-related safety and health concerns [26]. This phenomenon was termed the “Psychological Typhoon Eye” effect to describe the public’s psychological response, e.g., anxiety levels, safety and health concerns, to major emergencies and disasters.

To date, the “Psychological Typhoon Eye” effect has been detected after the Wenchuan earthquake [51], during the SARS epidemic [25] and in relation to lead-zinc mining risk [53]. Researchers have proposed three major possible explanations for this effect [52]. The first explanation is psychological immunization theory, which assumes that resistance to a stressful event is naturally acquired through repeated exposure [16]. People become desensitized by repeated exposure and can better prepare for stressful events. The second explanation is cognitive dissonance theory [8]. Cognitive dissonance is an uncomfortable psychological state in which the individual attempts to restore consistency or consonance by changing his or her beliefs and attitudes. When someone is at risk or in crisis, it is easier to change their beliefs and attitudes towards potential risk than to change their location [25, 26, 52]. Thus, people who are at the center of emergencies and disasters are presumably more likely than people living far away to believe that the risk is low and therefore continue to live nearby. The third explanation is the gap between experiencing/involving and imagining [25, 52], in which people in the center have a more accurate estimate of the risks based on real experience and involvement.

To date, few empirical studies has tested these explanations. However, all the explanations suggest that the influence of the level of exposure to an unfortunate event on mental health problems may be mediated by subjective risk perceptions. Risk perceptions are intuitive risk judgments [39] that include “the process of collecting, selecting, and interpreting signals about uncertain impacts of events, activities, or technologies” ([45], p.1049). A meta-analysis by Sheeran and his colleagues showed that risk perceptions have a close association with people’s health behavior [36].

According to protection motivation theory (PMT [29];), health attitudes and behavior depend on two key psychological factors of risk perception, including one’s perceived threat due to the risk and coping efficacy with regard to the ability to cope with the risk. Perceived threat consists of estimates of the chance of contracting a disease (perceived vulnerability) and estimates of the seriousness of a disease (perceived severity). Coping efficacy refers to beliefs about whether responses are available and effective in averting the threat (response efficacy) and whether people and groups can effectively respond to the risk and protect themselves from the hazard (self-efficacy).

To a great extent, the three explanations for the “Psychological Typhoon Eye” effect emphasize the role of coping efficacy in risk perceptions. The essence of psychological immunization is an increase in coping efficacy. With repeated exposure, individuals develop new patterns of coping to deal with the crisis. These patterns become an integral part of their repertoire of problem-solving responses and increase the likelihood that these individuals will deal more or less realistically with future hazards. In this way, the satisfactory resolution of one crisis increases resistance to subsequent adverse experiences [16]. Similarly, the essence of the gap between experiencing and imagining is that people in the center have high response efficacy and self-efficacy when they have a large amount of embodied experience or involvement compared with those without experience or involvement. Additionally, cognitive dissonance theory emphasizes that after applying the cognitive strategies of rationalization (i.e., restoring consonance), the coping efficacy of people in the center is strengthened. Among the three explanations, coping efficacy may be viewed as an internal mental indicator of psychological immunization. Cognitive dissonance and experience act as two pathways to enhance people’s coping efficacy. The former is a cognitive pathway and the latter is a behavioral pathway.

### Current study

The goal of this research was twofold. The first goal was to examine the robustness of the “Psychological Typhoon Eye” effect during the COVID-19 epidemic: the closer people are to the “center” of the epidemic (i.e., the higher the exposure level), the less serious their mental health problems are. To our knowledge, two studies have confirmed the “Psychological Typhoon Eye” effect with regard to the level of exposure to epidemics and mental health problems. These studies examined the relationship between the level of exposure and anxiety levels [51] and epidemic-related safety and health concerns [26]. In this study, we assessed mental health problems using a questionnaire adapted from the Psychological and Behavioral Questionnaire for SARS [9]. The questionnaire was designed to reflect the psychological state of the population during severe public health emergencies. It consists of five dimensions, i.e., depression, neurosism, phobia, compulsion-anxiety, and hypochondriasis. Compared to the two studies stated above, this study investigated broader facets of mental health problems rather than one specific aspect.

The second goal was to investigate the mechanism of the “Psychological Typhoon Eye” effect. As stated before, even though some possible mechanisms have been proposed, none of them have been verified by empirical studies. We draw on protection motivation theory to formulate a theoretical model of how the exposure level during the COVID-19 epidemic influences mental health problems.

**Fig. 1:**
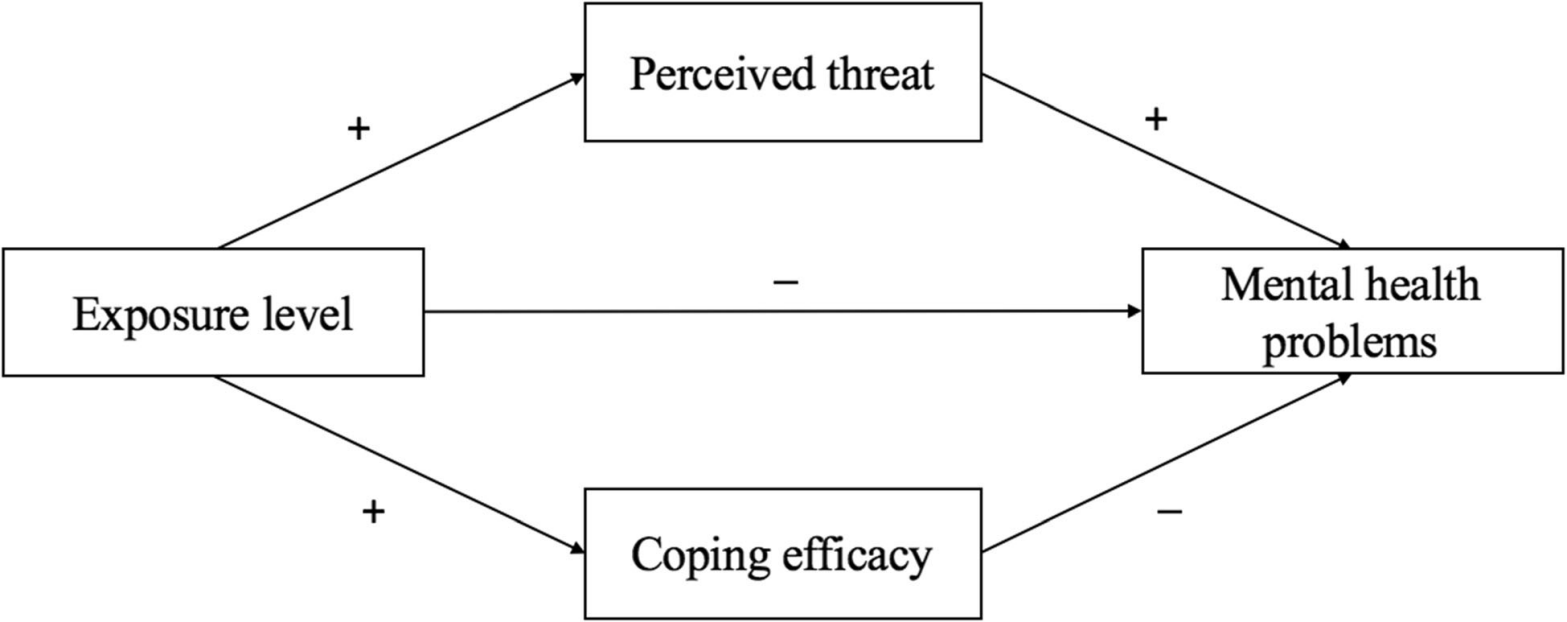
Proposed model of exposure level, risk perception and mental health problems

According to protection motivation theory, we hypothesized that the association between the exposure level during the COVID-19 epidemic and mental health problems was mediated by both individuals’ perceived threat of COVID-19 risk and their coping efficacy (see Fig. 1). More importantly, we hypothesized that the valence of the mediating effects was distinct. Both perceived threat and coping efficacy are positively correlated with the exposure level. However, perceived threat, which tends to aggravate mental health, is positively correlated with mental health problems. This hypothesis is based on evidence from SARS studies and COVID-19 studies. These studies showed that the relatively high perceived threat (severity and vulnerability) of SARS/COVID-19 played a pivotal role in the development of fear for the pandemic [31] or psychological distress [5, 6, 48] and increased the odds of individuals having a high level of depressive symptoms 3 years later [27].

In contrast, we hypothesized that coping efficacy, which tends to buffer mental health, is negatively correlated with mental health problems. This hypothesis is based on the fact that numerous studies have indicated that self-efficacy is an effective factor to cope with a crisis and buffer psychological distress [34]. A cross-sectional study of 415 respondents in a community health care setting showed that mental health status was negatively correlated with coping strategies, which can increase self-efficacy [38]. A systematic review article [19] found that psychological distress was prevalent among Ebola survivors, whose coping strategies included engagement with religious faith, Ebola survivor associations and involvement in Ebola prevention and control interventions. All of these coping strategies are beneficial to enhance self-efficacy and response efficacy to relieve psychological distress. Additionally, both qualitative and quantitative studies suggest that social support is an effective coping strategy for psychological distress [33] because it can promote self-efficacy [30, 50].

To achieve the two aforementioned purposes, we conducted a survey in 31 provincial-level administrative divisions of China during 3–13 March 2020. Our first hypothesis is that a “Psychological Typhoon Eye” effect exists between the level of exposure to epidemics and mental health problems. The second hypothesis is that there are two parallel routes between the exposure level and mental health problems. Specifically, perceived threat mediates the positive relationship between the exposure level to epidemics and mental health problems, while coping efficacy mediates the negative relationship between them. In other words, coping efficacy could account for the “Psychological Typhoon Eye” effect.

## Methods

### Participants

The online survey platform Wenjuanxing (https://www.wjx.cn) was employed to conduct this study during an eleven-day period (3–13 March 2020). The platform is a usable platform for user studies [20, 44, 49]. In total, 3459 participants from 31 provincial-level administrative divisions took part in the survey. The data of 471 participants who did not complete the survey seriously (average answer time less than 200 ms per question or answering repetitively for every question) were excluded. The final number of effective samples was 2987. This study was approved by the School of Sociology and Psychology Academic Committee, Central University of Finance and Economics. It takes around 10 mins to complete all questionnaires in this study, and participants received five RMB after their participation.

### Measurements

#### Mental health problems

The Mental Health Questionnaire was adapted from the Psychological and Behavioral Questionnaire during SARS [9], which was designed to reflect the psychological state of the population under severe public health emergencies. The adaptations made the items specifically applicable to COVID-19. Twenty-five items were categorized into five dimensions: depression (α =0.93; e.g., “I am easily fatigued and have difficulty recovering”), neurosism (α = 0.91; e.g., “I am interested in nothing”), phobia (α = 0.82; e.g., “I avoid going to hospitals or other crowded areas as much as possible and wear a mask when meeting people”), compulsion-anxiety (α = 0.93; e.g., “I have symptoms including rapid heartbeat, sweating and blushing”), and hypochondriasis (α =0.80; e.g., “I worry about being infected when I have related symptoms”). All the items were measured on 4-point scales from 0 to 3 according to the level of emotion (none, mild, moderate and severe) or frequency of behavior (occasionally, sometimes, often, always). We averaged the scores to obtain a score for every dimension (possible score range: 0–3). We averaged the ratings to obtain the scores for each dimension and the overall mental health score (α =0.969).

#### Exposure level

The accumulative number of confirmed cases was regarded as an indicator to evaluate the severity of the COVID-19 epidemic compared with other epidemic indicators (e.g., accumulative number of deaths, incidence rate, case fatality rate; see details in Table 1). All epidemic data were acquired from the official website of the National Health Commission on March 2nd, 2020, and this website is the most authoritative website for information on the epidemic during the COVID-19 in China. This study used the accumulative number of confirmed cases to represent the exposure level during COVID-19, see details in Table 2.

**Table 1:** Correlations among epidemic severity indicators during COVID-19

	1	2	3
1. accumulative number of confirmed	–		
2. accumulative number of deaths	1.00***	–	
3. incidence rate	0.99***	0.99***	–
4. case fatality rate	0.85***	0.85***	0.85**

**p < 0.01, ***p < 0.001

**Table 2:** Accumulated confirmed cases in different provinces on March 2, 2020

Province	Sample size	Number of cases	Province	Sample size	Number of cases
Hubei	399	67,217	Guangxi	83	252
Guangdong	294	1350	Shaanxi	33	245
Henan	252	1272	Yunnan	22	174
Zhejiang	85	1213	Hainan	4	168
Hunan	71	1018	Guizhou	15	146
Anhui	77	990	Tianjin	108	136
Jiangxi	63	935	Shanxi	155	133
Shandong	248	758	Liaoning	72	125
Jiangsu	162	631	Hongkong	3	100
Chongqing	33	576	Jilin	59	93
Sichuan	78	538	Gansu	20	91
Heilongjiang	38	480	Xinjiang	7	76
Beijing	187	414	Neimeng	39	75
Shanghai	93	338	Ningxia	23	74
Hebei	131	318	Taiwan	1	41
Fujian	132	296			

#### Perceived threat

The Perceived Threat Questionnaire was self-constructed based on the model of risk perception by Slovic [39]. This questionnaire was designed to reflect perceived vulnerability and perceived severity during the outbreak of COVID-19. A total of six items were used to measure perceived threat initially. All the items were measured on a 5-point scale from 1 (strongly disagree) to 5 (strongly agree). Item descriptions, and reliability and validity of variables can be seen in Tables 3 and 4. One item “I follow the official information released by the National Health Commission frequently” was removed due to its loading below 0.70 [14], so five items were used to represent perceived threat in final structural model. The discriminant validity results according to the Fornell-Larcker criterion are shown in Table 4.

**Table 3:** Items, and validity assessments of perceived threat and coping efficacy

Items	loading	VIF^a^
**Perceived threat (Cronbach’s Alpha: 0.82, CR**^**b**^**:0.85, AVE**^**c**^**:0.51)**		
“I think the COVID-19 epidemic in China is very serious”	0.77	1.85
“I think the COVID-19 epidemic is very serious abroad”	0.74	2.32
“I am concerned about the increase of imported COVID-19 cases”	0.73	2.10
“I think I am very close to the epidemic in Wuhan”	0.80	1.35
“I have a great deal of uncertainty about when the epidemic will end”	0.78	1.70
“I continue to closely monitor the information released by the authorities”	0.35	1.23
**Coping efficacy (Cronbach’s Alpha: 0.91, CR:0.94; AVE:0.78)**		
“I think the COVID-19 epidemic will be effectively controlled”	0.88	2.61
“I am optimistic about the situation of this epidemic”	0.88	2.61
“I believe that I can effectively deal with the COVID-19 epidemic”	0.88	2.55
“I believe we can effectively deal with the COVID-19 epidemic”	0.91	2.99

^a^VIF: variance inflation factor

^b^CR: composite reliability

^c^AVE: average variance explained

**Table 4:** Discriminant validity using Fornell-Larcker criterion

	Coping efficacy	Perceived threat
Coping efficacy	0.88	- ^a^
Perceived threat	0.07	0.71

^a^Not available

#### Coping efficacy

The Coping Efficacy Questionnaire was adapted from the Perceived Coping Efficacy Questionnaire used by Kim, Sherman and Updegraff [21], which was designed to reflect the participants’ belief that they and their groups could effectively protect themselves from the threat of Ebola. The adaptations made the items specifically applicable to COVID-19. Coping efficacy in the present study involves self-efficacy and response efficacy, and the four items are “I think the pneumonia epidemic will be effectively controlled”, “I am optimistic about the situation of this epidemic”, “I believe that I can effectively deal with the pneumonia epidemic” and “I believe we can effectively deal with the pneumonia epidemic”. The first two items mainly reflect response efficacy, while the last two items mainly reflect the self-efficacy. Four items were measured on a 5-point scale from 1 (strongly disagree) to 5 (strongly agree), and all of items have high reliability and validity, see details in Tables 3 and 4.

#### Covariates

The following covariates were included in the current study: age (under 18 years old, 2.8%, 18–25 years old, 36.4%; 26–30 years old, 23.7%; 31–40, 23.8%; 41–60, 12.6%; over 60, 0.6%), gender (female, 59.3%; male, 40.7%), income (below ¥1000, 19.4%; ¥1000–¥3000, 22.9%; 3000-¥5000, 24.4%; ¥5000–¥7000, 17.0%; ¥7000–¥10,000; 10.8%; over ¥10,000, 5.5%), educational level (junior high school or below, 24.6%; senior high school, 26%; bachelor’s degree, 39.4%; master’s degree and higher, 9.9%) and occupation (student, 22.3%; sales, 11.3%; production, 8.6%; administration/logistics, 5.9%; customer service, 4.5%; research and development, 4.4%; marketing/public relations, 4.0%; teacher, 3.1%; human resources, 2.9%; management, 2.8%; finance/audit, 2.6%; office work, 2.6%; professional, 2.1%; consulting, 0.6%; others, 22.0%).

### Data analysis

Data was analyzed using SPSS 21.0, and structural models among exposure levels, perceived threat, coping efficacy and mental health problems were used by partial least squares structural equation modeling (PLS-SEM) in SmartPLS 3.3 (Smart PLS GmbH). PLS-SEM has often been recommended for data analysis in the case of non-normal data [14]. In this study, original number of cases in the 31 provincial regions had great variances, and it doesn’t conform to a normal distribution. For example, Hubei Province had 67,217 accumulated cases of COVID-19 in 2 March, while the accumulated cases in other 30 provincial regions were under 1500, see details in Table 2. Significance testing at the 0.05 level (two-tailed) in PLS-SEM were generated by using 5000 subsamples.

## Results

### Exposure level and mental health problems

**Fig. 2:**
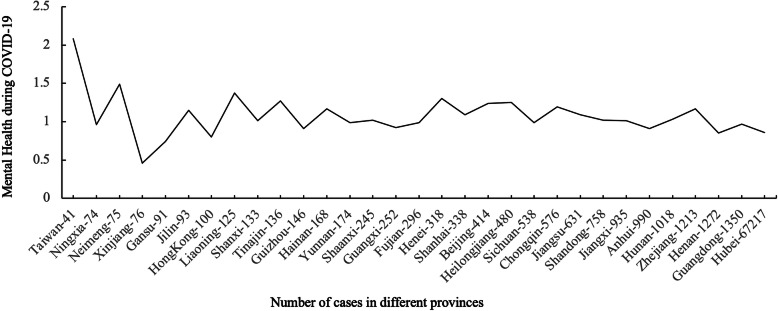
Exposure level and mental health problems in 31 provinces

The Fig. 2 illustrates the relationship between exposure level and mental health problems in 31 different provinces in China during COVID-19. Correlation analysis showed that exposure level (number of COVID-19 cases) was negatively related to mental health scores of people in 31 provinces in China, *r* = − 0.09, *p* < 0.001.

### Exposure level, risk perception and mental health problems

The correlations among the exposure level, risk perception and mental health problems during COVID-19 are presented in Table 5. The exposure level was negatively related to mental health problems, *p*< 0.001. Moreover, perceived threat was positively correlated with mental health problems, and coping efficacy was negatively related to mental health problems, *ps* < 0.001.

**Table 5:** Correlations among exposure level, risk perception and mental health problems during COVID-19

	1	2	3	4
1.Exposure level	–			
2.Perceived threat	0.18**	–		
3.Coping efficacy	0.11**	0.20**	–	
4.Mental health problems	−0.09**	0.30**	−0.27**	–

**p < 0.001

**Fig. 3:**
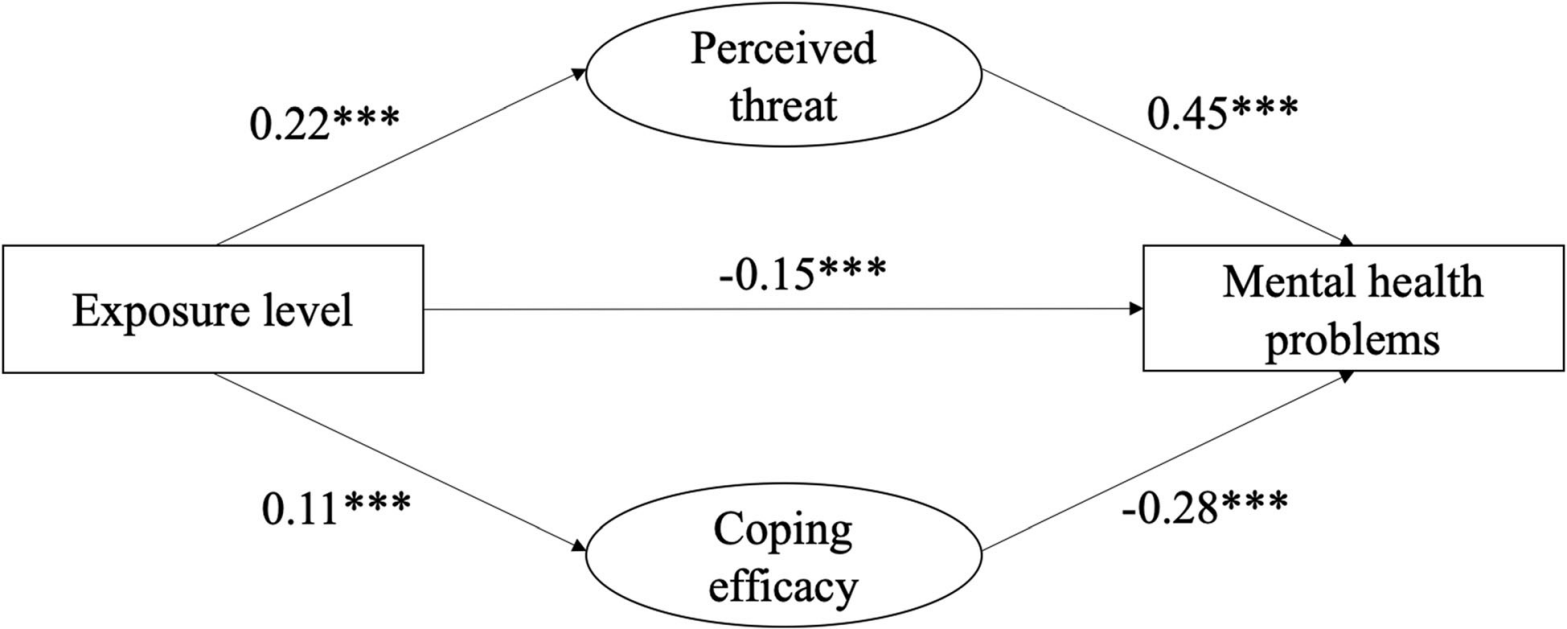
Models among exposure level, perceived threat and mental health problems during COVID-19

Furthermore, the mediating effects of risk perception between exposure levels and mental health problems were tested using the PLS-SEM in SmartPLS. We generated 5000 bootstrapping subsamples from the original data set (*N* = 2987). Table 6 displays the direct and indirect effects after controlling for age, gender, income, educational level, and occupation (as covariates). The model explained 23.4% variance in mental health problems. As shown in Fig. 3, the exposure level exerted a significant indirect effect on public mental health via perceived threat and coping efficacy.

**Table 6:** Indirect and direct effects in structural model

Relationships between variables		β	*t*	*p*
Direct Effects	Exposure level →Mental health problems	− 0.15	10.43	< 0.001
	Perceived threat →Mental health problems	0.45	31.70	< 0.001
	Coping efficacy→ Mental health problems	−0.28	19.45	< 0.001
	Exposure level →Perceived threat	0.22	11.33	< 0.001
	Exposure level→ Coping efficacy	0.11	6.53	< 0.001
Indirect Effects	Exposure level→ Perceived threat → Mental health	0.10	10.60	< 0.001
	Exposure level→ Coping efficacy→ Mental health	−0.03	6.27	< 0.001

## Discussion

The present study examined whether and how the level of exposure to COVID-19 in China influenced mental health problems. The results showed that the exposure level to COVID-19 in China was negatively associated with mental health problems related to COVID-19. Specifically, the higher the exposure level to COVID-19, the better mental health was. More importantly, this study is the first to reveal the mechanism by which the level of exposure to COVID-19 is linked to mental health problems related to COVID-19. Specifically, perceived threat mediated the positive relationship between them, while coping efficacy mediated the negative relationship between them.

Our finding of less serious mental health problems related to COVID-19 for people with higher exposure levels to COVID-19 in China confirms the psychological typhoon eye effect rather than the ripple effect. This finding is consistent with several previous studies [25, 51] of public emergency events in China, which reported that proximity to the center of the epidemic or devastated area was negatively related to the public’s irrational panic and mental distress. Additionally, this finding is in accordance with a counterintuitive phenomenon in which intense states, such as emergency events, may abate more quickly than mild states because intense states trigger psychological processes that are designed to attenuate them [11]. This phenomenon is an instance of a more general phenomenon known as the region-β paradox, which demonstrates that the relation between time and distance is nonmonotonic since people tend to use faster modes of transportation to cover longer distances [11].

According to our findings, the underlying mechanism is that coping efficacy mediates the negative relationship between the level of exposure to COVID-19 and mental health problems. In other words, it is the coping efficacy that accounts for the psychological typhoon eye effect. Theoretically, as mentioned above, all explanations in previous studies, including the psychological immunization theory, cognitive dissonance theory, and the theory of the description–experience gap [25, 52], have emphasized the essential and potential role of efficacy. In the framework of psychological immunization theory, people in areas of high exposure would acquire more self-efficacy to cope with the epidemic because people become desensitized after repeated exposure. In this sense, their immunization ability is improved. Similarly, in the framework of the description-experience gap theory, a more accurate estimate of the risks based on real experience and involvement increases the sense of control and efficacy. In the framework of cognitive dissonance theory, individuals apply the cognitive strategy of rationalization to achieve a state of consonance to restore a sense of self-control and self-efficacy. Generally, people fail to anticipate the extent to which their psychological immune systems will hasten the recovery from disaster or major negative events, which is termed immune neglect [12, 46]. As such, the triggered psychological process, i.e., the cognitive strategy of rationalization, helps individuals reduce negative states more quickly, which in turn subjectively enhances self-efficacy. In summary, all three explanations in previous studies directly or indirectly emphasize the role of efficacy, which is a pivotal factor in our model.

The mediating role of coping efficacy can be easily understood in the context of collectivist Chinese culture. In collectivist countries, when the public is exposed to the center of an epidemic or devastated area, a high level of coping efficacy is stimulated [17, 21, 40]. Appropriate response efficacy at the national level provides sufficient information and psychological support for the public, which in turn increases coping efficacy. Additionally, many empirical studies have shown that self-efficacy is an effective factor to buffer psychological distress (e.g., [3, 19, 50]) and that response efficacy is positively correlated with health behavior (e.g., [15, 43]).

This study also showed that the perceived threat of COVID-19 was positively related to mental health problems related to COVID-19, which is consistent with previous evidence in relation to SARS (e.g., [5, 6, 48]). Furthermore, perceived threat mediated the positive relationship between the level of exposure to COVID-19 and mental health problems related to COVID-9. Specifically, this finding can explain the ripple effect (i.e., the higher the exposure level, the stronger the mental distress). However, considering the specific results of this study (i.e., the negative relationship between the exposure level and mental health problems), perceived threat may be a suppressor in the negative relationship. Taken together, the two pathways suggest that the two mechanisms work simultaneously, but the valence of the indirect effects is reversed. In summary, coping efficacy rather than perceived threat could explain the psychological typhoon eye effect.

Regarding the psychological typhoon eye effect and the ripple effect, we preliminarily speculate which effect dominates may be a result of balance between perceived threat and coping efficacy. They can be seen as two sides of seesaw. When perceived threat is too high and coping efficacy is too low, people may experience the overwhelming fear and hopelessness [47]. When coping efficacy is too high and perceived threat is too low, people may underestimate the risk and not adopt coping strategies to avert the threat. Only when perceived threat is high enough to arouse coping efforts, and is nearly comparable to coping efficacy, both of them function greatly and they may dominate the seesaw alternatively. Depending on which one is higher between coping efficacy and perceived threat, mental health problems related to the stressful emergency demonstrate the psychological typhoon eye effect or the ripple effect. When coping efficacy is higher than perceived threat, the related mental health problems may demonstrate the psychological typhoon eye effect; when coping efficacy is lower than perceived threat, the mental health may demonstrate the ripple effect. Our data were collected on 3–13 March 2020 when the number of new cases decreased to single digits and scientific prevention and control as well as orderly resumption of work and production was promoted. Perceived threat should be slightly lower than coping efficacy. Therefore, the psychological typhoon eye effect was seen in our study. Our assumptions can be used to understand some phenomena. For example, although cyberchondria is generally regarded to be negative, in the case of COVID-19, it might have made people understand the threat of the situation [7]. However, when constantly seeing news and reports highlighting the threat of COVID-19, people will start to suffer from stress and anxiety [7]. We can imagine that by seeking news and reports highlighting coping efficacy, people’s mental health states may be better when their coping efficacy is increased to be higher than perceived threat. Taken together, emergency management like COVID-19 demands dynamic balance between perceived threat and coping efficacy [7, 47]. However, our speculations are very preliminary and remains to be tested empirically in future studies.

## Conclusion

Overall, this study confirmed the psychological typhoon eye effect during the outbreak of COVID-19 in China and demonstrated the mediating role of coping efficacy and perceived threat between exposure to COVID-19 and mental health problems. Our findings suggest that policy makers and psychological workers should provide enough psychological services to low-risk areas as the high-risk areas. An important means of alleviating mental health problems is to improve coping efficacy.

However, our findings may be restricted to people during an epidemic who live in collectivist countries. It remains unclear whether our findings are applicable in other countries or after the epidemic. China is a typical collectivist country. People in the center of outbreaks in China obtain intensive and extensive social support from the government, enterprises, individuals and society. Therefore, coping efficacy can play an important mediating role. It is not clear whether our findings hold true in other countries. More studies in other countries are needed to confirm our findings.

In addition, our results cannot exclude the possibility that people in the center of emergencies and disasters are occupied with coping, and therefore some types of mental health problems emerge only after the epidemic. Some longitudinal studies have indicated that SARS survivors still had elevated stress levels and worrying levels of psychological distress even after 1 to 4 years [23, 41]. Medical staff who performed MERS-related tasks showed the highest risk of posttraumatic stress disorder symptoms even after time had elapsed [24]. Therefore, although we observed a negative correlation between the level of exposure and mental health problems, we do not suggest stopping or reducing psychological assistance to people in the center of the outbreak. Psychological workers and policy makers should provide appropriate psychological services depending on the level of exposure and epidemic stage.

Finally, this study did not directly test the associations between coping efficacy and three explanations including psychological immunization, cognitive dissonance and description-experience gap. Thus, these specific claims regarding their associations are more speculative, which would need to be addressed empirically in future work. The relationship between perceived threat and coping efficacy is also fascinating which is beyond the scope of this article. More future studies are needed to examine the relationship between them.

## Data Availability

The raw data supporting the conclusions of this manuscript will be made available by the authors to any qualified researcher.
